# Advantage from massive parallel sequencing of *RYR1* and *CACNA1S* in diagnostics of malignant hyperthermia susceptibility

**DOI:** 10.1186/1471-2253-14-S1-A13

**Published:** 2014-08-18

**Authors:** Dorota Fiszer, Nickla A Fisher, Ian M Carr, Marie-Anne Shaw, Philip M Hopkins

**Affiliations:** 1 Leeds Institute of Biomedical & Clinical Sciences, School of Medicine, University of Leeds, Leeds, LS9 7TF, UK; 2 Malignant Hyperthermia Investigation Unit, St James’s University Hospital, Leeds, LS9 7TF, UK

## Background

Mutations in *RYR1* (ryanodine receptor type 1) are linked to the majority of malignant hyperthermia (MH) families (75%) and some cases of Exertional Heat Stroke. Two *CACNA1S* variants associated with MH have been functionally characterised. Historically, because of the large size of the RYR1 gene, MH families were screened for 31 diagnostic *RYR1* variants only. Methods based on PCR and Sanger sequencing were used. The new opportunity arose since next generation sequencing techniques became available. With this technique sequencing is considerably quicker therefore even long gene sequences such as *RYR1* can be easily screened for variations.

## Material and methods

We used Next-Generation Sequencing to look for coding sequence non-synonymous variants of *RYR1* and *CACNA1S*. We applied two different target enrichment methods, Long-Range PCR and HaloPlex (Agilent), to be able to focus on particular regions of the genome.

## Results

In our study we included a group of 80 unrelated MHS individuals who were previously screened for diagnostic mutations in *RYR1* and were found negative. Another group consisted of discordant individuals, whose in vitro contracture test (IVCT) phenotype was at odds with their genotype for a familial diagnostic *RYR1* mutation. The third studied cohort (n=38) were Exertional Heat Stroke patients, IVCT tested susceptible or normal.

## Conclusions

Our results illustrate that Next Generation Sequencing provides broad genetic information quicker and at lower cost than conventional approaches. To improve understanding of malignant hyperthermia genetics, screening of susceptible individuals only for a limited number of diagnostic *RYR1* variants is not sufficient. Studying the full length of *RYR1* and *CACNA1S* coding sequence may also resolve some phenotype/genotype discordant cases. Finally, Next Generation Sequencing techniques provide opportunities for studying larger gDNA fragments or the whole genome, which may result in discovering some other genes linked to MH.

